# Thyroid hormone levels paradox in acute ischemic stroke

**DOI:** 10.1515/tnsci-2022-0289

**Published:** 2023-06-07

**Authors:** Chunhui Xie, Yi Jiang, Xiaozhu Shen, Mengqian Liu, Yiwen Xu, Wen Zhong, Zhonglin Ge, Mingyue Qian, Nan Dong, Chen Gong, Guanghui Zhang

**Affiliations:** Department of Neurology, The Affiliated Lianyungang Hospital of Xuzhou Medical University, Lianyungang, China; Department of Geriatrics, Lianyungang Hospital Affiliated to Jiangsu University (Lianyungang Second People’s Hospital), Lianyungang, China; Department of Geriatrics, Lianyungang Clinical College of Bengbu Medical College (Lianyungang Second People’s Hospital), Lianyungang, China; Department of Neurology, Lianyungang Hospital affiliated to Xuzhou Medical University, Lianyungang, China; Department of Neurology, Lianyungang Second People’s Hospital, Lianyungang, China; Department of Neurology, Suzhou Industrial Park Xinghai Hospital, Suzhou, China; Department of Geriatrics, Lianyungang Hospital Affiliated to Jiangsu University (Lianyungang Second People’s Hospital), Lianyungang, China

**Keywords:** acute ischemic stroke, thyroid hormones, prognosis, biomarkers

## Abstract

**Objective:**

Accumulating evidence has suggested that thyroid hormone levels affect the prognosis of acute ischemic stroke (AIS), but the results have been inconsistent.

**Methods:**

Basic data, neural scale scores, thyroid hormone levels, and other laboratory examination data of AIS patients were collected. The patients were divided into excellent and poor prognosis group at discharge and 90 days after discharge. Logistic regression models were applied to evaluate the relationship between thyroid hormone levels and prognosis. A subgroup analysis was performed based on stroke severity.

**Results:**

A number of 441 AIS patients were included in this study. Those in the poor prognosis group were older, with higher blood sugar levels, higher free thyroxine (FT4) levels, and severe stroke (all *p* < 0.05) at baseline. Free thyroxine (FT4) showed a predictive value (all *p* < 0.05) for prognosis in the model adjusted for age, gender, systolic pressure, and glucose level. However, after adjustment for types and severity of stroke, FT4 showed insignificant associations. In the severe subgroup at discharge, the change in FT4 was statistically significant (*p* = 0.015), odds ratio (95% confidence interval) = 1.394 (1.068–1.820) but not in the other subgroups.

**Conclusions:**

High-normal FT4 serum levels in patients with severe stroke receiving conservative medical treatment at admission may indicate a worse short-term prognosis.

## 1 Introduction

Stroke was the second leading cause of death worldwide after ischemic heart disease. At the same time, 101 million people in the world suffered from stroke and 6.55 million people dead from stroke [1]. Stroke was also a major cause of death in China, with approximately 3.94 million new cases of stroke and 2.19 million stroke-related deaths each year. As of 2019, there have been about 28 million cases of stroke; 24 million of these were cases of acute ischemic stroke (AIS) [2]. From 2013 (2.28%) to 2019 (2.58%), the prevalence of stroke in China increased by 13.2%, with an annual increase rate of 2.2% [3].

With age, changes occur in all bodily systems, including the endocrine system. The thyroid function is particularly important due to its central role in metabolism, thermogenesis, and immunity, among others, as well as because of its contribution to most common chronic age-associated diseases, especially cardiovascular disorders [4,5,6]. The prevalence of thyroid dysfunction increases with the aging of the global population [6,7]. The relationship between thyroid hormones and cardiovascular health has attracted considerable research attention [8,9]. Observational findings have revealed that not only the abnormal range of thyroid hormones but also their normal range is involved in the occurrence and development of cardiovascular diseases [10,11]. However, no consensus has been achieved on the optimal target level of thyroid hormones in acute ischemic stroke patients. The results of previous studies on the association between acute thyroxine levels and the clinical outcomes in AIS patients are contradictory [12]. For example, a study including 129 patients with AIS indicated that thyroid hormone levels were not an independent predictor of stroke outcome [13]. Another study focused on the association of anterior pituitary hormones with stroke indicated that thyroid hormones were not associated with stroke prognosis [14]. The Rotterdam Study revealed that free thyroxine (FT4) levels in elderly subjects were positively associated with atherosclerosis throughout the whole disease spectrum, independently of cardiovascular risk factors [15]. On the other hand, subclinical hypothyroidism (SCH) and low FT4 levels have been associated with increased carotid intima-media thickness in recent studies [16,17,18]. In a retrospective study of 221 AIS cases and 182 non-AIS cases, detailed clinical data showed that lower triiodothyronine (FT3) concentrations measured at admission were predictive of poor neurological function within three months [19]. Another investigation prospectively recruited 563 intravenous thrombolysis (IVT) patients from five stroke centers in China and established that subclinical hyperthyroidism (SHyper) was an independent predictor of 3-month poor outcome and mortality [20]. We can thus speculate that low FT3, high FT4, and low thyroid-stimulating hormone (TSH) can predict a poor prognosis in patients with acute ischemic stroke. Thus, we conducted this study to investigate whether thyroid hormone levels within the normal reference range are associated with poorer functional outcome in AIS patients and to explain the underlying mechanisms.

## 2 Methods

### 2.1 Study design and population

This study was ambispective cohort study. Patients diagnosed with acute ischemic stroke with standardized treatment at Lianyungang Hospital affiliated to Xuzhou Medical University were consecutively included. The complete study cohort consisted of two parts. One part retrospectively was collected from September 1, 2019, to October 21, 2021, and the other prospectively was from October 21, 2021, to December 31, 2021.

Standardized treatment was given to patients according to guidelines [21] for respiratory support, blood pressure, temperature and glucose management, and antiplatelet therapy but does not include thrombolysis and endovascular therapy.

Patients were enrolled in this study if they met the following criteria: (1) age ≥18 years old; (2) onset within 48 h; (3) presence of acute ischemic lesions in anterior circulation, which were confirmed by imaging methods (magnetic resonance imaging or computed tomography); and (4) received standardized treatment.

Exclusion criteria for this study were: (1) intracranial hemorrhage or mass lesion; (2) with severe infection or septic shock; (3) liver or renal failure; (4) incomplete laboratory, clinical or follow-up data; (5) thyroid dysfunction (normal reference ranges were those routinely used at Lianyungang First People’s Hospital Laboratory: for TSH, 0.56–5.91 mIU/L; for FT4, 7.9–17.0 pmol/L; and for FT3, 3.8–6.47 pmol/L); and (6) received thrombolysis or mechanical thrombectomy.

### 2.2 Data collection

Baseline clinical information of all enrolled patients was collected from the database, including age, sex, systolic blood pressure (SBP), diastolic blood pressure (DBP), the National Institutes of Health Stroke Scale (NIHSS), history of hypertension, diabetes (DM), atrial fibrillation (AF), stroke or coronary heart disease (CHD), current smoking (any usage of cigarette per day in the past 30 days), and drinking (drinking more than 100 mL [alcohol content >50% liquor] per day on average and drinking for more than 1 year; abstaining from drinking for more than 1 year is not). NIHSS score on admitted was determined by two trained neurologists. Laboratory findings at admission included TSH, FT4, FT3, blood glucose (BG), glycated hemoglobin (HbA1c), total cholesterol (TC), triglycerides (TG), low-density lipoprotein cholesterol (LDL), high-density lipoprotein cholesterol (HDL), homocysteine (HCY), blood urea nitrogen (BUN), and creatinine (Cr). We calculated BUN to creatinine (BUN/Cr) ratio, because BUN/Cr was identified as an potential predictor indicator for poor outcomes of patients with stroke [22]. The trial of Org 10172 in Acute Stroke Treatment (TOAST) classification was divided into large-artery atherosclerosis (LAA), cardioembolic (CE), small artery occlusion (SAO), stroke of other determined etiology (SOE), and stroke of undetermined etiology (SUE) [23]. Patients were assessed for stroke severity by NIHSS score, with ≤4 being mild stroke and >4 being severe stroke [24].

### 2.3 Functional outcome and follow-up

The functional outcome was assessed by the modified Rankin Scale (mRS) at discharge and 90 days after onset. 90-day mRS was determined by two trained operators via telephone. An excellent outcome was defined as mRS 0–2.

### 2.4 Blood thyroid function tests measurement

Within 24 h after admission, all patients underwent venous blood drawing for routine laboratory after overnight fast. Serum TSH, FT4, and FT3 were measured by the chemiluminescence immunoassay (Beckman Coulter DXI800).

### 2.5 Statistical analysis

The analyses were done with R version 4.2.3 (R Core Team 2022). Kolomogorov–Simirnov test was used to identify the normality of data. The normal distribution data were analyzed by independent sample *t* test, and the non-normal distribution data were analyzed by Mann–Whitney *U* test. Fisher’s exact test or the chi-square test was used to compare categorical variables as appropriate. Statistical significance was determined as a bilateral test.

Univariate logistic regression analysis was used to explore the association between baseline information and prognosis. Multivariate logistic regression analysis model was used to determine the relationship between the thyroid hormone levels and functional outcome in acute ischemic stroke and controlled confounding factors. Three multivariate logistic regression models were developed. Model 1 was without adjusting any covariates; model 2 was adjusted for age, gender, and variables that were significant in the univariate regression except for neural scales. Model 3 added neural scale scores to model 2.

All subjects were divided into two subgroups: NIHSS ≤4 subgroup and NIHSS >4 subgroup. Multivariate logistic regression was used to evaluate the relationship between thyroid hormone levels and stroke prognosis in subgroups (the variables are the same as those in model 4 but do not include NIHSS). *p* < 0.05 (bilateral) is defined as a statistically significant difference.


**Ethical approval:** The research related to human use has been complied with all the relevant national regulations, institutional policies and in accordance with the tenets of the Helsinki Declaration, and has been approved by the authors’ institutional review board or equivalent committee. Ethical approval for this study was obtained from the ethics committees of Lianyungang Hospital affiliated to Xuzhou Medical University (No. KY-20210917001-01).
**Informed consent:** Informed consent has been obtained from all individuals included in this study.

## 3 Results

### 3.1 Study population and clinical characteristics

**Figure 1:**
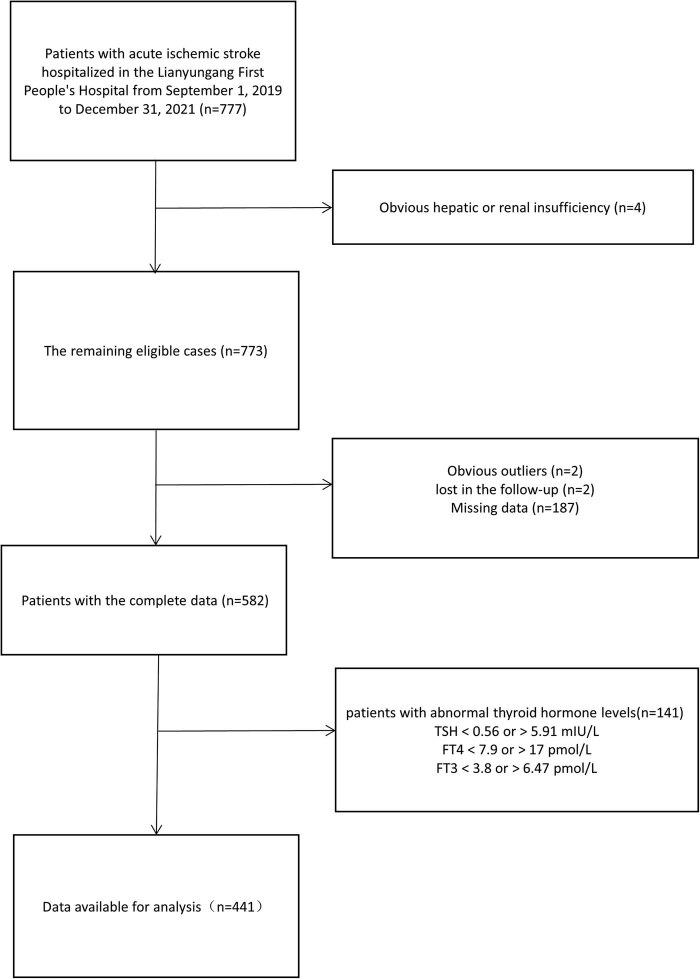
Subjects screening flowchart. Abbreviations: TSH, thyroid-stimulating hormone; FT4, free thyroxine; FT3, free triiodothyronine.

The study population was 777 patients with acute ischemic stroke who received conventional medical therapy. After screening with exclusion criteria, 441 patients were finally determined as the population meeting the study criteria (Figure 1). Due to the small sample size of SOE and SUE in TOAST classification, they were combined into other group for easier statistical analysis. As shown in Table 1, compared with the group with poor functional outcomes, gender, DBP, smoking, drinking, atrial fibrillation, hypertension, CHD, TSH, FT3, HbAlc, TC, TG, HDL, LDL, Hcy, BUN, Cr, and BUN/Cr had a similar distribution in the group with excellent outcomes (all *p* ≥ 0.05) whether mRS was scored at discharge or 90 days later. People with higher mRS were older and had higher NIHSS scores, higher BG levels, higher FT4 levels, and higher proportion of DM and LAA (all *p* < 0.05).

**Table 1:** Baseline characteristics of patients

		mRS at discharge			90-Day mRS		
	Total (*n* = 441)	Excellent prognosis (*n* = 334)	Poor prognosis (*n* = 107)	*p*	Excellent prognosis (*n* = 343)	Poor prognosis (*n* = 98)	*p*
**Parameter**							
	163 (37.0)	121 (36.2)	42 (39.3)	0.653	124 (36.2)	39 (39.8)	0.589
Age (years)	66.00 [57.00, 72.00]	65.50 [57.00, 71.00]	68.00 [58.00, 77.00]	0.038	66.00 [57.00, 71.50]	67.50 [58.00, 77.00]	0.061
SBP (mmHg)	146.00 [137.00, 156.00]	145.00 [137.00, 154.75]	150.00 [137.00, 164.00]	0.017	146.00 [137.50, 155.00]	150.00 [137.00, 162.75]	0.054
DBP (mmHg)	85.00 [79.00, 92.00]	85.00 [79.00, 92.00]	85.00 [80.00, 92.50]	0.841	85.00 [79.00, 92.00]	84.50 [80.00, 92.75]	0.797
**Medical history**							
Smoking	132 (29.9)	101 (30.2)	31 (29.0)	0.898	103 (30.0)	29 (29.6)	1.000
Drinking	117 (26.5)	91 (27.2)	26 (24.3)	0.635	92 (26.8)	25 (25.5)	0.897
AF	35 (7.9)	26 (7.8)	9 (8.4)	0.997	26 (7.6)	9 (9.2)	0.760
DM	114 (25.9)	76 (22.8)	38 (35.5)	0.013	79 (23.0)	35 (35.7)	0.016
Hypertension	281 (63.7)	212 (63.5)	69 (64.5)	0.941	220 (64.1)	61 (62.2)	0.822
Stroke	81 (18.4)	56 (16.8)	25 (23.4)	0.164	59 (17.2)	22 (22.4)	0.301
CHD	34 (7.7)	27 (8.1)	7 (6.5)	0.755	27 (7.9)	7 (7.1)	0.981
**Biochemical**							
TSH (mmol/L)	1.71 [1.17, 2.49]	1.71 [1.19, 2.50]	1.67 [1.10, 2.46]	0.482	1.72 [1.19, 2.51]	1.60 [1.07, 2.40]	0.327
FT4 (mmol/L)	12.19 [11.02, 13.40]	12.04 [10.90, 13.18]	12.58 [11.50, 13.91]	0.001	12.07 [10.92, 13.28]	12.54 [11.42, 13.82]	0.007
FT3 (mmol/L)	4.65 [4.32, 5.00]	4.64 [4.31, 5.00]	4.66 [4.42, 4.98]	0.818	4.65 [4.31, 5.00]	4.65 [4.42, 4.96]	0.965
BG (mmol/L)	5.33 [4.68, 7.07]	5.19 [4.64, 6.28]	5.97 [5.06, 7.82]	<0.001	5.20 [4.64, 6.30]	5.97 [5.06, 8.18]	<0.001
HbA1c (mmol/L)	6.00 [5.60, 7.20]	6.00 [5.60, 7.00]	6.20 [5.60, 7.70]	0.173	6.00 [5.60, 7.05]	6.15 [5.60, 7.75]	0.242
TC (mmol/L)	4.55 [3.89, 5.27]	4.53 [3.90, 5.20]	4.60 [3.86, 5.54]	0.325	4.52 [3.89, 5.20]	4.65 [3.87, 5.55]	0.202
TG (mmol/L)	1.45 [1.04, 2.18]	1.43 [1.05, 2.08]	1.59 [1.00, 2.25]	0.523	1.43 [1.06, 2.09]	1.59 [0.97, 2.22]	0.848
HDL (mmol/L)	1.05 [0.88, 1.19]	1.04 [0.88, 1.19]	1.07 [0.94, 1.20]	0.183	1.04 [0.88, 1.19]	1.09 [0.94, 1.22]	0.092
LDL (mmol/L)	2.52 (0.69)	2.49 (0.65)	2.60 (0.79)	0.169	2.49 (0.66)	2.63 (0.77)	0.074
Hcy (μmol/L)	9.80 [8.10, 12.30]	9.70 [8.10, 12.30]	10.00 [8.05, 12.15]	1.000	9.70 [8.10, 12.30]	10.00 [8.03, 12.23]	0.997
BUN (mmol/L)	5.45 [4.50, 6.48]	5.48 [4.59, 6.47]	5.20 [4.30, 6.49]	0.400	5.46 [4.57, 6.47]	5.41 [4.32, 6.50]	0.533
Cr (mmol/L)	59.00 [49.20, 70.00]	59.20 [49.85, 70.25]	57.50 [46.35, 69.45]	0.269	59.20 [49.70, 70.20]	57.05 [46.62, 69.38]	0.279
BUN/Cr	0.09 [0.07, 0.11]	0.09 [0.07, 0.11]	0.09 [0.08, 0.11]	0.836	0.09 [0.07, 0.11]	0.09 [0.08, 0.11]	0.739
**NIHSS > 4**	113 (25.6)	36 (10.8)	77 (72.0)	<0.001	42 (12.2)	71 (72.4)	<0.001
**TOAST**				0.002			0.005
LAA	189 (42.9)	126 (37.7)	63 (58.9)		132 (38.5)	57 (58.2)	
CE	33 (7.5)	26 (7.8)	7 (6.5)		26 (7.6)	7 (7.1)	
SAO	206 (46.7)	172 (51.5)	34 (31.8)		175 (51.0)	31 (31.6)	
Other	13 (2.9)	10 (3.0)	3 (2.8)		10 (2.9)	3 (3.1)	

Note: abbreviations: systolic blood pressure (SBP), diastolic blood pressure (DBP), National Institutes of Health Stroke Scale (NIHSS), diabetes (DM), atrial fibrillation (AF), coronary heart disease (CHD), thyroid-stimulating hormone (TSH), free thyroxine (FT4), free triiodothyronine (FT3), blood glucose (BG), glycated hemoglobin (HbA1c), total cholesterol (TC), triglycerides (TG), low-density lipoprotein cholesterol (LDL), high-density lipoprotein cholesterol (HDL), homocysteine (Hcy), blood urea nitrogen (BUN) and creatinine (Cr), BUN to creatinine ratio (BUN/Cr), modified Rankin Scale (mRS), the Trial of Org 10172 in Acute Stroke Treatment (TOAST), large-artery atherosclerosis (LAA), cardioembolic (CE), small artery occlusion (SAO). Other defined as stroke of other determined etiology and stroke of undetermined etiology combined.

### 3.2 Univariable and multivariable analyses of functional outcomes at discharge and 90 days

A univariate analysis was conducted for each variable in the baseline table using a logistic regression. The resulting variables were defined as functional outcomes at discharge and 90 days, respectively. Variables with statistical significance are listed in Table 2. DM, FT4, BG, and NIHSS showed statistical significance at discharge and 90 days after discharge (all *p* < 0.05). When TOAST was used as a factor variable in the single-factor logistic regression model (LAA was used as a control), only SAO showed a correlation with the outcome variable (all *p* < 0.05). In addition, the SBP showed statistical significance at discharge (*p* = 0.029).

**Table 2:** Univariate logistic regression for functional outcomes

	OR	95% CI	*p*
**At discharge**			
Diabetes	1.87	1.167–2.995	0.009
SBP	1.014	1.001–1.026	0.029
BG	1.101	1.029–1.179	0.006
FT4	1.249	1.101–1.417	0.001
NIHSS > 4	21.246	12.314–36.659	<0.001
TOAST			
CE	0.538	0.222–1.308	0.172
SAO	0.395	0.246–0.637	<0.001
Other	0.6	0.159–2.258	0.45
**90-Day mRS**			
Diabetes	1.857	1.145–3.011	0.012
BG	1.107	1.032–1.186	0.004
FT4	1.195	1.051–1.359	0.006
NIHSS > 4	18.846	10.892–32.608	<0.001
TOAST			
CE	0.623	0.256–1.519	0.298
SAO	0.41	0.251–0.671	<0.001
Other	0.695	0.184–2.619	0.591

Note: A univariate analysis was conducted for each variable in the baseline table using a logistic regression. The resulting variables were defined as functional outcomes at discharge and 90 days, respectively. Meaningful variables were displayed in Table 2.

**Figure 2:**
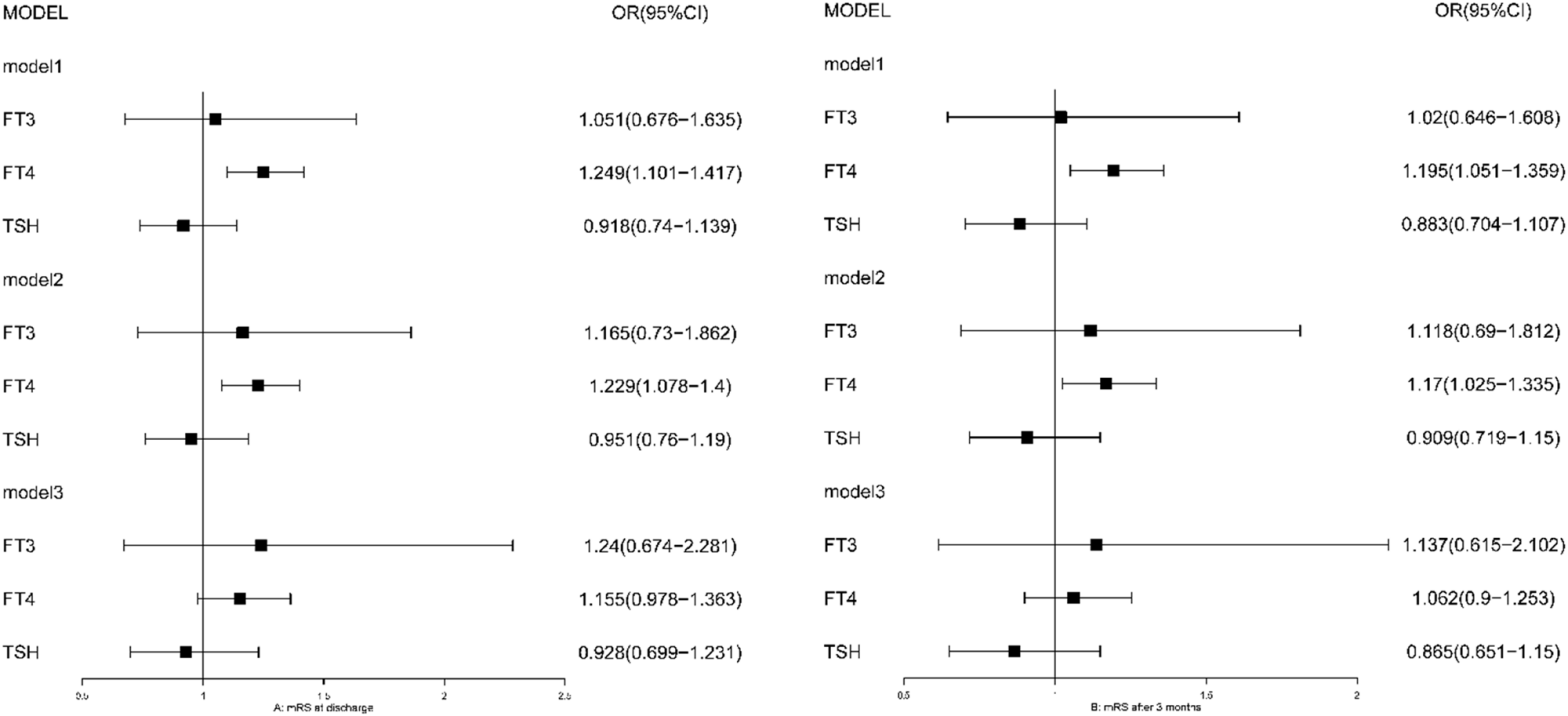
Forest plot about four logistic regression. Note: model 1 was without adjusting any covariates; model 2, additionally adjusted for age, Gender, SBP, and BG; model 3 added NIHSS and TOAST.

Two logistic regression models were used to evaluate the relationships between functional outcomes and thyroid hormone levels. The resulting variables were defined as functional outcomes at discharge and 90 days, respectively. FT4 was statistical significance in model 1 (no adjust), model 2 (additionally adjusted for age, sex, SBP, BG; to avoid interactions, BG was included in model 2 but DM not), and model 3 (with the addition of TOAST and NIHSS in model 2), the relationship between FT4 and functional outcome became not significant (all *p* > 0.05) (Figure 2). In model 3, NIHSS was statistical significance (all *p* < 0.05), but TOAST was not significant (all *p* > 0.05) (Table 3). In the two regression models, FT3 and TSH were not statistically significant (all *p* > 0.05), no matter how to adjust the covariates.

**Table 3:** Statistical value in model 3

	At discharge		After 90 days	
	OR (95% CI)	*p*	OR (95% CI)	*p*
NIHSS > 4	20.216 (11.339–36.041))	<0.001	17.339 (9.8–30.68)	<0.001
**TOAST**				
CE	0.272 (0.085–0.872)	0.028	0.399 (0.132–1.211)	0.105
SAO	0.589 (0.325–1.07)	0.083	0.617 (0.339–1.124)	0.115
Other	0.842 (0.165–4.294)	0.836	1.004 (0.203–4.969)	0.996

Note: The statistical values of the newly added variables NIHSS and TOAST in two models 3 were displayed.

### 3.3 Association between FT4 levels and functional outcomes in subgroups

On the group with NIHSS ≤4, none of the variables were found to be significant. In the population with severe stroke at admission, higher FT4 levels indicated higher mRS at discharge, *p* = 0.015, odds ratio (95% confidence interval) = 1.394 (1.068–1.820). But FT3 and TSH still were not significant (all *p* > 0.05) (Table 4).

**Table 4:** Multiple logistic regression for functional outcomes in NIHSS subgroups

	NIHSS >4		NIHSS ≤4	
	OR (95% CI)	*p*	OR (95% CI)	*p*
**At discharge**				
TSH	0.886 (0.596–1.316)	0.547	0.953 (0.635–1.428)	0.814
FT4	1.394 (1.068–1.82)	0.015	0.981 (0.775–1.242)	0.873
FT3	1.827 (0.654–5.103)	0.250	1.011 (0.45–2.27)	0.979
**After 90 days**				
TSH	0.817 (0.558–1.197)	0.300	0.894 (0.586–1.364)	0.604
FT4	1.186 (0.933–1.508)	0.164	0.923 (0.723–1.178)	0.521
FT3	1.552 (0.59–4.079)	0.373	0.948 (0.405–2.221)	0.903

Note: NIHSS was divided into two subgroups: NIHSS >4 group and NIHSS ≤4 group. TSH, FT4, and FT3 were evaluated again according to the method of building model 4. However, variables in model 4 no longer include NIHSS.

## 4 Discussion

Studies have shown that many systemic diseases, rather than thyroid-associated disorders, cause abnormal changes in serum thyroid hormone levels, in which thyroxine (T4) is not properly converted to triiodothyronine (T3), and inactive thyroid hormone T3 is accumulated in the body, known as euthyroid sick syndrome (ESS) [25]. ESS includes low-T3 syndrome, low-T3 and -T4 syndrome, high-T4 syndrome, and other abnormalities [26]. ESS has been observed in a variety of critical illnesses [27]. Common causes include severe infections, cranial trauma, respiratory failure, heart failure, starvation, surgery, and psychiatric disorders [28,29,30,31,32,33,34].

Thyroid hormones such as T3 and T4 have an irreplaceable role in the development, differentiation, and maturation processes of the brain tissue [35]. Thyroid hormones are secreted by the thyroid follicular epithelial cells. In peripheral blood, most T3 (80–90%) is converted from T4 by deiodinase [12]. In the blood circulation, some of the T4 is converted into T3 by deiodinase. T3 shows three- to five-fold greater activity than T4. Both hormones circulate in the peripheral blood in two forms: free and bound; the former enters the target cells and performs biological functions [36]. Interestingly, T3 and T4 have similar chemical structures and biological functions but have different associations with stroke prognosis [37,38]. In another investigation, patients with poor outcomes had significantly lower FT3 and serum total triiodothyronine (TT3) levels, and FT3/FT4 ratio, but higher FT4 levels [12]. A retrospective case–control study in patients with early-onset ischemic stroke without diabetes and hypertension revealed significantly lower levels of FT3 and FT4 in IS patients than in the controls [39]. In our research work, participants with higher mRS had higher FT4 levels. We further separated long-term and short-term prognoses and found that higher FT4 in patients with severe stroke at admission could indicate a worse short-term prognosis. No association between thyroid hormones and long-term prognosis of stroke was observed. Therefore, we speculated that the changes in FT4 in acute stroke are related mainly to the emergency response. We hypothesized that during emergency, increased synthesis of FT4 might have occurred, while no timely conversion of FT4 to FT3 was provided or was inhibited.

Thyroid hormones play a paradoxical role in AIS. The present research work on the relationship between thyroid hormone metabolic status and stroke prognosis is focused on the areas specified below.

### 4.1 SCH

SCH refers to a state in which TSH levels are higher than the normal, but those of free T3, T4, and total T3 and T4 are still in the normal range [27]. Acute ischemic stroke patients with SCH at admission were more likely to have excellent functional outcomes than those without SCH [40]. A significant protective association of SCH, with better outcomes and lower mortality after CIS, was found in another investigation [41]. Additionally, patients with acute ischemic stroke with high levels of TSH tended to have a better outcome [40,42]. Another study with 756 acute ischemic stroke patients recruited within seven days of onset established that the proportion of patients with excellent outcomes was significantly higher in the SCH group than in the control group on the 90th day [43]. Possible explanations for this association are ischemic preconditioning, reduced adrenergic tone, and hypometabolic state [30,36]. A study including 88 patients with cerebral infarction, most of which did not receive IVT, did not show a relationship between TSH and patient functional outcome [44], which is consistent with the conclusions of our work.

### 4.2 Subclinical hyperthyroidism

Subclinical hyperthyroidism is defined by reduced TSH levels in the presence of normal FT4 and FT3 values [45]. The low TSH levels of 199 patients in another previous investigation predicted poor clinical outcomes after endovascular thrombectomy in patients with anterior circulation ischemic stroke [46]. Furthermore, patients with subclinical hyperthyroidism had a higher risk of poor functional outcomes at 3 months after a stroke and a decreased rate of successful reperfusion after reperfusion therapy than patients in the euthyroid state [47]. Similar results were obtained in the present study. Although without a statistically significant difference, TSH in that earlier study was lower in the poor prognosis group (at discharge or at 90 days) than in the excellent prognosis group [42,47], which was not confirmed in our investigation.

### 4.3 Low-T3 syndrome

Low-T3 syndrome refers to decreased thyroid function and T3 level but normal T4 and TSH levels [48]. Reportedly, low FT3 values (<2.00 pg/mL) in 702 consecutive patients with acute stroke were independently associated with poor functional outcome and mortality at 3 months after stroke onset [49]. The results of 11 studies including a total number of 3936 enrolled patients with acute stroke showed that patients with a poor prognosis had lower T3 levels [12]. However, no similar results were obtained in our work. The median value of FT3 in our study population was 4.65 pmol/L, and the median value of FT3 in both the excellent and poor prognosis groups was close to this level.

### 4.4 High-T4 state

High-T4 state serum T4 levels may actually be elevated early in acute illness due to either the acute inhibition of type 1 iodothyronine deiodinases or an increase in thyroxine-binding globulin (TBG) levels [50,51]. This state is most often observed in the elderly and in patients with psychiatric disorders [52]. As the duration of illness increases, nondeiodinative pathways of T4 degradation increase, and serum T4 levels return to the normal range [53]. This may explain why higher FT4 in patients with severe stroke at admission is associated with a worse short-term prognosis in our study. A study in 2017 restricted to the LAA population showed an association between FT4 and 14-day mRS, but the NIHSS was not included in the analysis. In this study, we further explored the relationship between stroke severity and short-term prognosis. We speculated that the elevated FT4 was consistent with the elevated BG at the onset, which may be related to a short-term stress state.

The results of this study suggest that higher FT4 is associated with worse short-term prognosis of stroke but not with long-term prognosis. We speculate that it may be that fluctuations in thyroid hormones in stroke accelerate disease progression or have other adverse effects. Subsequently, the hormone levels slowly return to normal and the adverse effects disappear or are reduced. By measuring FT4 levels in patients, doctors earlier predicted the development of stroke severity. And this phenomenon may provide new ideas for treatment to delay the progression of stroke. We speculate that future studies could find new thresholds with AIS for thyroid hormone levels, doctors intervene in thyroid hormone levels in AIS patients who in the short term of the attack for the delay of stroke progression and improve prognosis.

### 4.5 Limitations

Nevertheless, the present study has some limitations. This was a single-center retrospective study, which might have selection bias. Since the population in this study was of Asian descent, the results may not be applicable to other ethnic groups. Also, the secondary outcomes, including mortality and hemorrhage, need to be evaluated in future large-scale studies.

## 5 Conclusions

High-normal FT4 serum levels in patients with severe stroke receiving conservative medical treatment at admission may indicate a worse short-term prognosis.
